# Olefin–Styrene Copolymers

**DOI:** 10.3390/polym8110405

**Published:** 2016-11-19

**Authors:** Nunzia Galdi, Antonio Buonerba, Leone Oliva

**Affiliations:** Dipartimento di Chimica e Biologia “Adolfo Zambelli”, Università degli Studi di Salerno, via Giovanni Paolo II, 84084 Fisciano, Italy; gnunzia@unisa.it (N.G.); abuonerba@unisa.it (A.B.)

**Keywords:** Ziegler-Natta, homogeneous catalysis, stereoregular polymer, regiocontrol, crystallinity

## Abstract

In this review are reported some of the most relevant achievements in the chemistry of the ethylene–styrene copolymerization and in the characterization of the copolymer materials. Focus is put on the relationship between the structure of the catalyst and that of the obtained copolymer. On the other hand, the wide variety of copolymer architecture is related to the properties of the material and to the potential utility.

## 1. Introduction

Ethylene and styrene are very popular monomers, each one individually responsible for some tens of megatons of polymer materials per year. However, their reactivity towards most initiators and catalysts is so different that their copolymerization seemed impossible until about 25 years ago.

Since the way to copolymerization was paved by homogeneous polyinsertion catalysis, an extraordinary variety of structures appeared in the macromolecular literature that is not easy to find for other couples of monomers. Beside the so-called pseudorandom copolymer of commercial interest, the further ethylene–styrene arrangements along the polymer chain represent a paradigm of the possible statistics and of the structure–property relationship.

## 2. Ethylene–Styrene Copolymerizations

### 2.1. Homogeneous Catalysis

**Figure 1:**
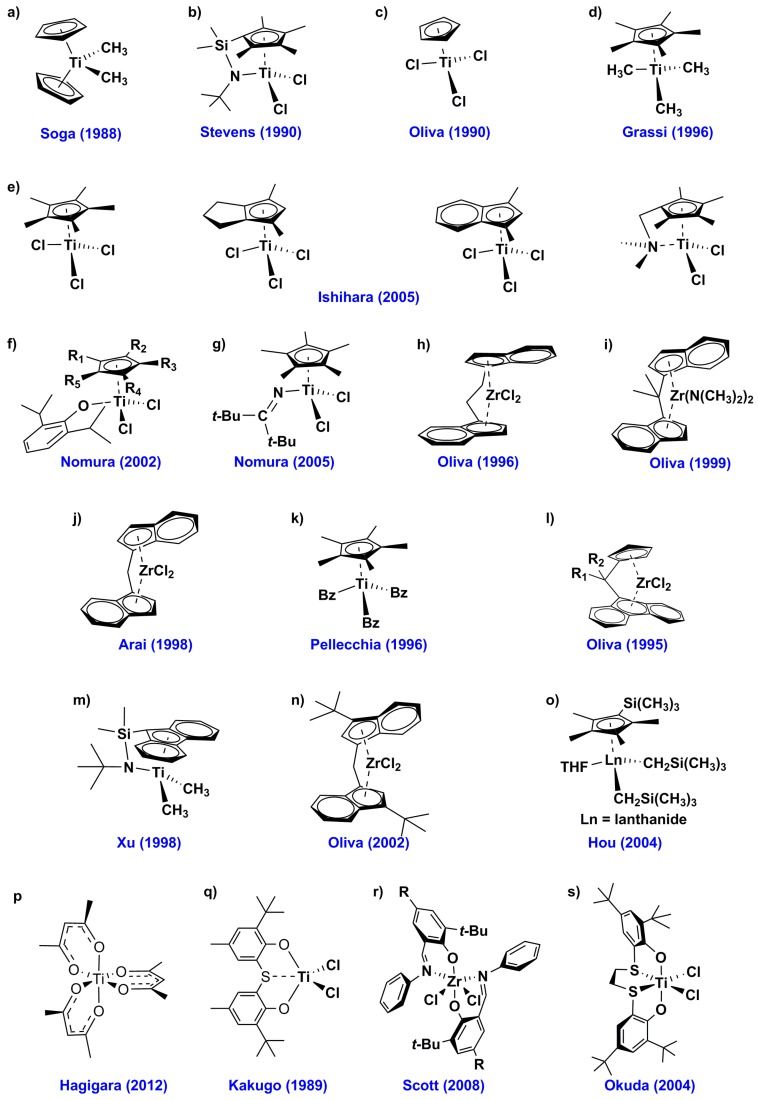
Complexes for ethylene–styrene copolymerization.

The history of ethylene–styrene copolymers begins around 1990. Actually, previous attempts to produce these copolymers both by free radical and by heterogeneous Ziegler–Natta catalysis were unsuccessful, because the very relevant difference in the reactivity between the two monomers affords, at most, polyethylene with occasional styrene units (Figure 1a) [1].

The key of the achievement was the homogenous polyinsertion catalysis based on transition metals in group 4. In particular, a Dow patent by Stevens et al. [2] claimed the successful synthesis of ethylene–styrene copolymers lacking head-to-tail styrene homosequences, which the authors called pseudorandom interpolymers. The main components of these catalytic systems are the constrained-geometry complexes (see Figure 1b as an example) activated by methylalumoxane (MAO). The materials obtained following the recipes of the patent show elastomeric behavior even below the normal ambient temperature, and have been commercialized since 1999 [3].

Besides the classic exploitation, the sulfonation of ethylene–styrene pseudorandom copolymers affords proton-conducting membranes with possible application for fuel cells [4]. 

Practically contemporaneous to the above discussed patent, is the report on the synthesis of similar macromolecules in the presence of monocyclopentadienyl titanium trichloride and other soluble titanium (IV) compounds activated by MAO (Figure 1c) [5,6]. In the copolymer chains, the styrene molar content is always less than 50% with substantial absence of styrene–styrene sequences. In fact, with these catalytic systems the copolymer chains are produced in a mixture with syndiotactic polystyrene, from which they can be easily separated. Possibly, this is the consequence of the presence of different catalytic species with different oxidation states of the titanium, being generally accepted that in the active site for syndiotactic polystyrene synthesis the metal is in the oxidation state +3 as a consequence of the titanium reduction in the reaction mixture (Figure 1d) [7].

Ishihara et al. formulated a discussion on the matter by reporting further evidences about the coexistence of different active centers in catalytic systems based on some half-titanocene compounds (Figure 1e) [8]. In their paper, the authors evoked the synergic action of a couple of ethylene molecules in order to have insertion into the titanium–benzyl bond of the polystyrene growing chain.

Later efficient systems based on (cyclopentadienyl)(aryloxy) titanium(IV) have been deeply investigated by the Nomura group (Figure 1f) [9,10,11]. The same author achieved the living ethylene–styrene copolymerization by using a (cyclopentadienyl)(ketimide) titanium (IV) complex activated by MAO (Figure 1g) [12]. Even in the materials obtained with this catalyst were detected NMR signals due to pseudorandom sequences, but actually the styrene content is at most 12 mol % and any inference about the statistics of copolymerization seems unreliable [12]. The ethylene–styrene (ES) copolymers obtained with the above-cited catalysts can show some crystallinity only at low styrene content, whenever the ethylene homosequences are long enough to organize themselves into a lattice. Consequently, the X-ray diffraction pattern is very similar to that of the polyethylene. On the other hand, the capability to crystallize is almost completely lost if the styrene content reaches 20% in mol (about 50% in weight). In correspondence with this value, the copolymer can behave as a thermoplastic elastomer with good elastic recovery [13]. With respect to the elastomeric character, two groups of researchers of Dow Chemical Co. reported in-depth studies on the properties of the ES interpolymers as a function of the composition [14,15,16].

**Figure 2:**
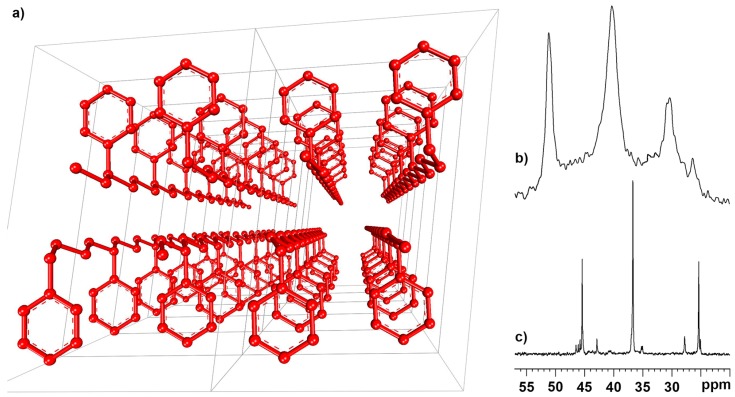
Crystalline structure of isotactic poly(ethylene-*alt*-styrene) copolymer (**a**); cross-polarization magic-angle spinning (CP-MAS) ^13^C-NMR (**b**); and solution ^13^C (**c**) spectra of the aliphatic region of the same copolymer.

The copolymerization of ethylene and styrene can be achieved also in the presence of *ansa*-zirconocene catalysts to produce macromolecules still lacking styrene homosequences [17]. With these catalytic systems, when the styrene unit content rises close to 50 mol %, one can observe the occurrence of an isotactic structure that again induces some crystallinity, with a typical X-ray diffraction pattern, due to the regular alternation of the two monomer units (Figure 1h) [18]. The melting point, determined by differential scanning calorimetry (DSC), is around 135 °C, very close to that of high-density polyethylene (HDPE). The study of the X-ray spectra of oriented fibers of these copolymers indicates a zigzag planar chain conformation with phenyl groups oriented perpendicularly to the chain axis (see Figure 1i and Figure 2a) [19], whereas it is well known that the isotactic polystyrene chains are arranged as helices into the lattice [20,21]. This alternate structure can be achieved at low polymerization temperature in the presence of *ansa*-zirconocene catalytic complex having a small bite angle, whereas zirconocenes with an increased angle are able to give alternate ES copolymers even at room temperature and higher (compare Figure 1h,j) [22,23]. A further insight into the conformational arrangement of the alternate copolymer chains in the crystalline lattice is given by the solid-state cross-polarization magic-angle spinning (CP-MAS) ^13^C-NMR (Figure 2b). The comparison of the chemical shifts suggests a more extended conformation in the solid state with respect to that in solution (Figure 2c), in agreement with the conclusions of the diffractometric analysis [19].

**Figure 3:**
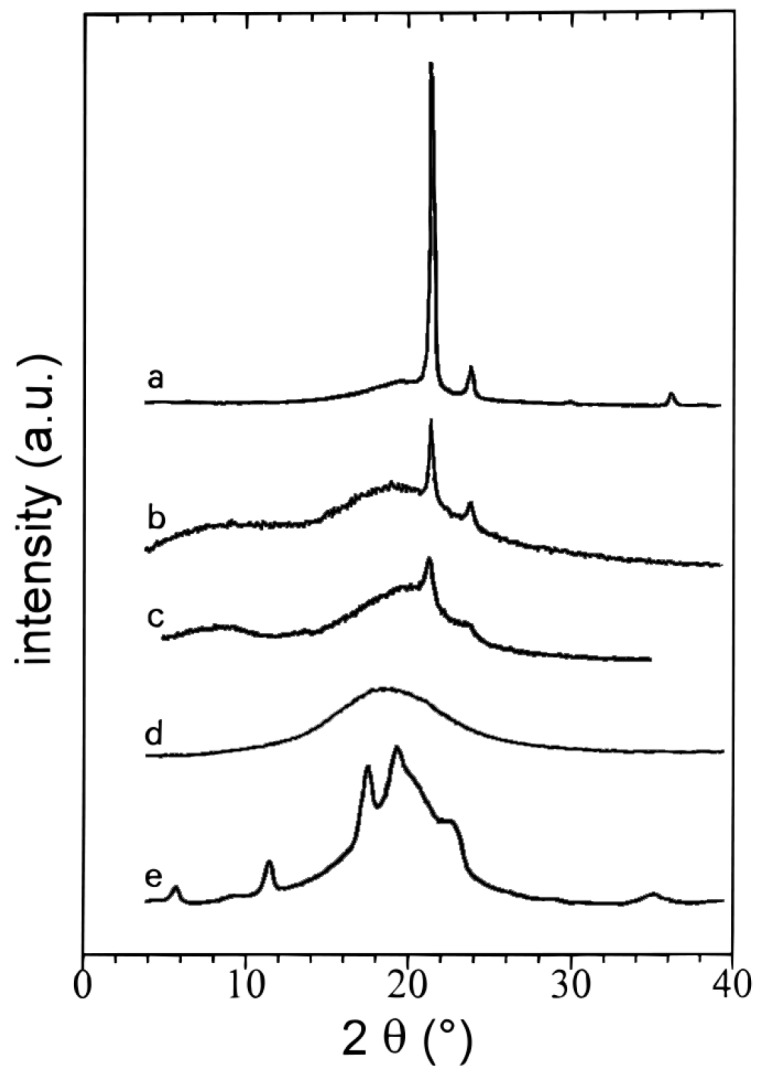
Wide-angle X-ray diffraction spectra of: high-density polyethylene (HDPE) sample (**a**) and ethylene–styrene (ES) copolymers at increasing styrene content (**b**–**e**). Reproduced from [24] with authorization of American Chemical Society.

**Figure 4:**
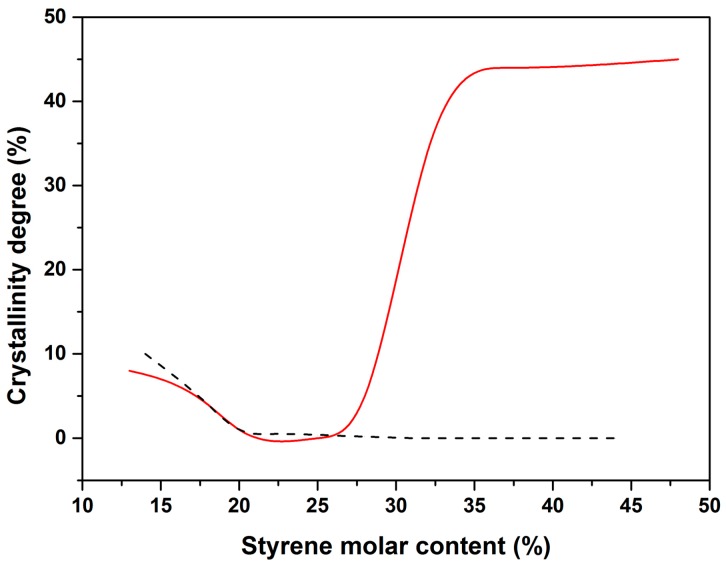
Crystallinity degree of ES copolymers vs. styrene molar content: copolymers obtained with *ansa*-zirconocene catalyst (**solid line**); copolymer obtained with half-titanocene catalyst (**dash line**).

It could be instructive to follow the behavior of a series of ethylene–styrene copolymers with increasing styrene content by comparing those obtained through stereoselective catalysis with those synthesized without stereocontrol (see Figure 3 and Figure 4) [24]. At low styrene content, both kinds of copolymer show some crystallinity due to the ethylene homosequences (Figure 3). The crystallinity disappears around 20 mol % styrene content and, as consequence, elastomeric behavior takes place. The further increase of the presence of styrene units does not change the properties of the atactic copolymer in a relevant manner; on the contrary, the copolymer from a stereoselective catalyst exhibits some crystallinity, of the kind above-described (Figure 3), by approaching the 50 mol % styrene content (Figure 4).

It is worth noting, additionally, that an alternate ES copolymerization was obtained also in the presence of MAO-free non-stereoselective catalyst based on monocyclopentadienyltitanium, affording an atactic structure unable to crystallize (Figure 1k) [25].

### 2.2. Mechanistic Aspects

By coming back to the catalytic aspects of the synthesis of the stereoregular alternating copolymers it is worth mentioning the key role played by the regiochemistry of the styrene insertion. Usually for the polymerization processes, the regiochemical aspects are considered for the defects (head-to-head and tail-to-tail placements) induced into the chain as consequence of the lack of regiocontrol [26]. Actually, in the metal-catalyzed polyinsertion the regiochemistry can play some more complex roles by driving the chain structure, as is the case of styrene and, as recently reported by Resconi et al., also for propene polymerization [27].

**Scheme 1:**
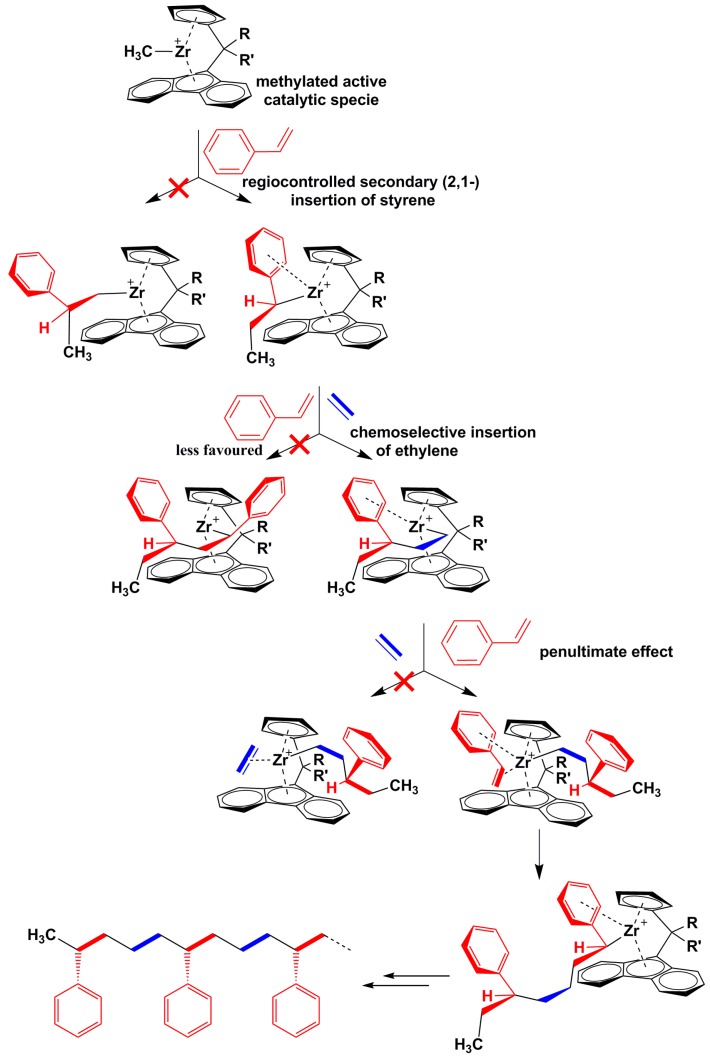
Regio- and stereocontrolled alternating copolymerization of ethylene and styrene leading to formation of isotactic poly(ethylene-*alt*-styrene) copolymer (ethylene and styrene units in the polymer chain are respectively drawn in blue and red).

For styrene, the use of a ^13^C-enriched cocatalyst and NMR analysis evidenced that the aromatic monomer inserts into the Zr–C bond with formation of a metal–benzyl bond (see Scheme 1 and Figure 1l) [28]. After this event, further styrene insertion should be slow, and consequently the relative reactivity of the ethylene increases. This picture becomes more complicated if one observes the triads’ composition, whose statistical analysis shows a clear a penultimate unit effect. Possibly, the aromatic ring of styrene’s next-to-last unit interacts with the metallic center, making it less prone to coordinate and, therefore, to insert ethylene. This mechanistic picture, first proposed on the basis of experimental kinetic studies [29], was restated through independent computer-aided calculations [30,31].

With regard to the stereochemistry, the geometry of the catalyst preferentially selects one enantiotopic face of the prochiral monomer, so producing isotactic E-*alt*-S copolymer. Notably, the isotacticity is observed as well with the C_S_ symmetric catalysts known to polymerize propene to syndiotactic polypropylene. In this case, in accordance with the mechanism of the migratory insertion, the strict alternation of ethylene and styrene incorporation leads the prochiral monomer to always insert at the same site and consequently with the same enantioface (see Figure 1l,m) [29,32].

### 2.3. Terpolymers

**Scheme 2:**
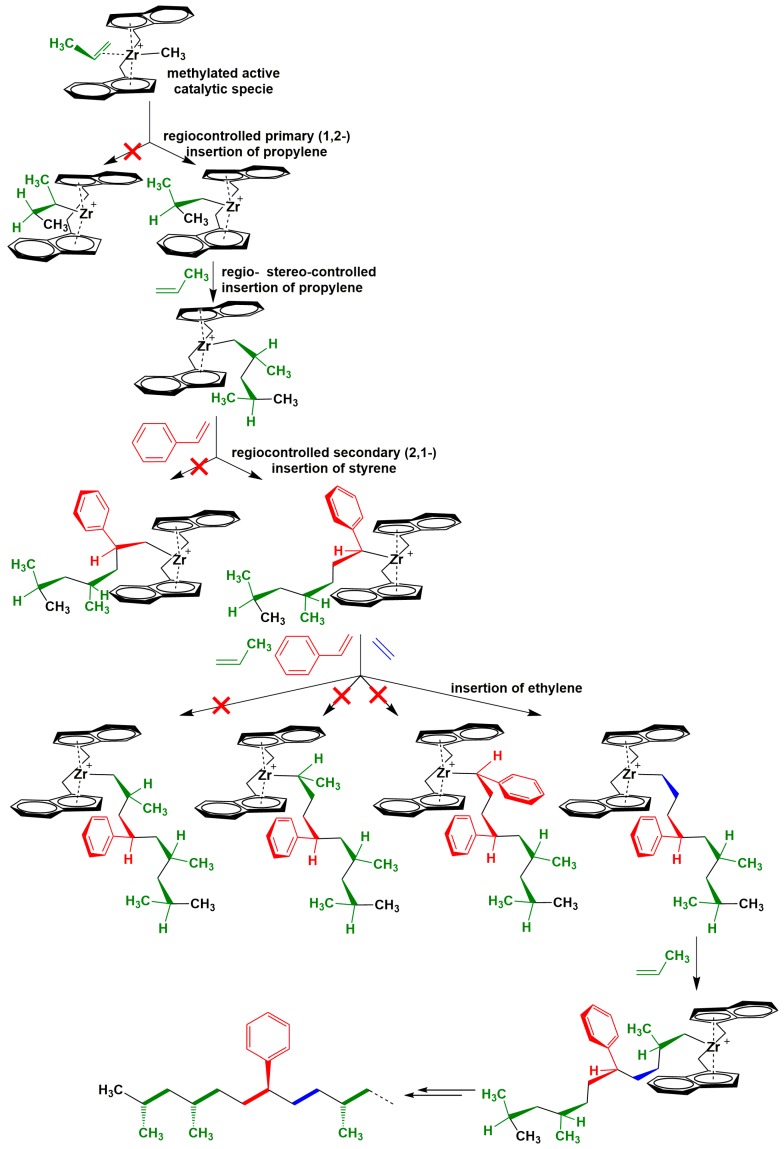
Terpolymerization of propylene, styrene, and ethylene leading to isolated ethylene–styrene couple-jointing isotactic polypropylene sequences (propylene, ethylene and styrene units in the polymer chain are respectively drawn in green, blue and red).

The regiochemical evidences above-recalled are the key to understand why these catalytic systems are unable to copolymerize styrene with 1-alkenes. In fact, 1-alkenes are known to insert in primary mode—that is, with formation of a metal–methylene bond—and this kind of insertion possibility is forbidden after the styrene insertion, due to the hindrance of the two tertiary carbons that should link together (see Scheme 2). In effect, one could suppose that through its insertion, the styrene generates a sleeping site unable to further insert styrene or 1-alkene. This mechanistic interpretation suggested the idea of using small amounts of the simplest olefin (ethylene) to reawaken the site after the styrene insertion, so allowing further propylene polyinsertion. Actually, copolymers were synthesized where ethylene–styrene couples joint isotactic polypropylene sequences (see Scheme 2) [33,34].

**Figure 5:**
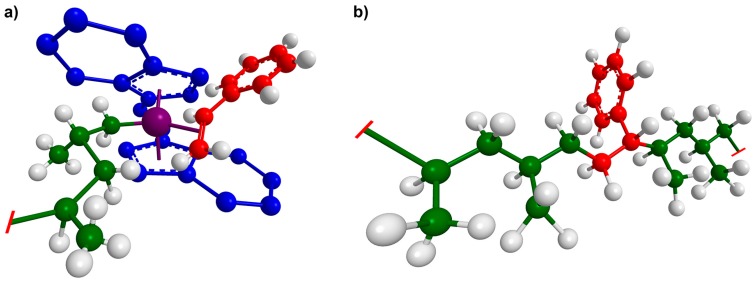
Enantiofacial selectivity of *ansa*-zirconocene catalyst (complex in blue with the metal in purple and hydrogen atoms omitted merely for clarity) with C_2_-symmetry for propylene and styrene copolymerization (**a**); and the resulting zigzag planar polymer chain (**b**) (propylene and styrene units in the polymer chain are respectively drawn in green and red).

Besides the novelty content of the material, being essentially an isotactic polypropylene containing functionalizable aromatic rings, in this synthesis was also hidden the opportunity of disclosing some stereoselective aspects of the styrene insertion, particularly relating the chirality of the site (R, R or S, S) to the preferred monomer enantioface (*re* or *si*). The investigations to enlighten this relationship were performed with the support of small molecules as models. Through NMR analysis, these studies allowed one to conclude that the (R, R) *ansa*-zirconocenes prefer the enantioface *re* of the styrene, when the insertion is secondary (see Figure 5) [35,36]. So, the polymer chain in planar zigzag conformation has the phenyl group on the opposite side of the plane with respect to the methyl groups.

The potential interest of this new material was explored by employing *p*-methylstyrene in place of styrene. Through metalation, the methyl group of the aromatic ring becomes a reactive initiation center for anionic polymerization and can be used as point of attack for the growth of grafts. Some graft copolymers, such as *i*-PP-*g*-PS, have been synthesized and, when tested as compatibilizer, they work well in reducing the phase separation of the polystyrene–polypropylene polymer blends [37,38,39]. Besides, it was possible to evaluate the relationship between the copolymer feature and its ability to compatibilize, thanks to the wide flexibility of the synthetic pathway, which allows tuning the graft density as well as the graft length.

### 2.4. Block-Wise Copolymers

The regiochemical induction responsible of the alternating ES copolymerization disappears when the coordinative framework of the *ansa*-zirconocene complex bears a bulky substituent like the *t*-butyl group (Figure 1n) [40]. In this case, the steric hindrance, overcoming the electronic factors, compels the styrene monomer to the primary insertion and the resulting ES copolymer shows a block-wise structure [40]. Possibly, as a consequence of the different regiochemistry, the mechanistic picture previously described [29] does not work more, and after a primary styrene insertion further primary insertions are allowed, giving rise to polystyrene sequences. 

**Figure 6:**
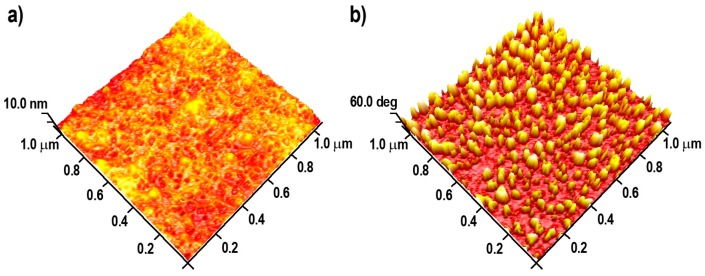
Height (**a**) and phase contrast (**b**) tapping-mode atomic force microscopy (TM-AFM) micrographs of an iPS-*b*-iP(E-*alt*-S) block copolymer with 23 wt % of iPS in the polymer backbone. Reproduced from [41] with permission from The Royal Society of Chemistry.

A very different block-wise structure of ES copolymers has been recently obtained through the quasi-living polymerization that can be achieved with the usual catalysts at low temperature. So, at 0 °C a judicious choice of the comonomers feed allows the synthesis of ES copolymer structures, where the isotactic polystyrene sequence is jointed to the alternating ES sequence [41]. Depending on the comonomers’ composition, these materials in the solid state organize themselves into different nanostructures, which can be observed through atomic force microscopy (AFM) techniques. As a matter of fact, they have been detected as either circular nanodomains with about a 30 nm diameter (due to self-assembling of the copolymer chains) or bicontinuous nanostructured phases, depending on the block lengths. Figure 6 shows a tapping-mode atomic force microscopy (TM-AFM) micrograph of a sample with 23 wt % of *i*-PS showing circular rigid domains (with an average diameter of 27 nm) embedded in a soft polymer matrix.

Moreover, the possibility of synthesizing block copolymers of ethylene and syndiotactic polystyrene was disclosed by Hou and coworkers [42], who using a scandium-based cationic catalyst (Figure 1o), and more recently by Hagigara et al. [43] (Figure 1p), who obtained a mixture of polymer materials from which they isolated macromolecules with long syndiotactic polystyrene sequences jointed to long polyethylene sequences. This achievement was gained by using a Ti(III)-based catalyst and could be seen as a further piece of the puzzle, where the oxidation state of the titanium is related to the copolymerization ability.

### 2.5. Non-Metallocene Systems

The interest towards the homogeneous polyinsertion catalysis was reawaken by the so-called post-metallocene catalysts, based mainly on pseudo-octahedral complexes of metals of group 4, activated by methylaluminoxane. Such catalysts have also been tested in ES copolymerization. Precursors of this new wave were Kakugo et al., who described the synthesis of the isotactic ethylene-*alt*-styrene copolymer by using the octahedral complex 2,2′-thiobis(4-methyl-6-*t*-butylphenoxy) titanium dichloride (Figure 1q) [44]. This milestone remains controversial because attempts to reproduce it by other researchers were unsuccessful [45].

More recently, Scott et al. [46] reported that the popular Fujita catalyst based on salicylaldiminate zirconium dichloride (Figure 1r) is unable to copolymerize ethylene and styrene due to the generation of dormant sites after styrene insertion. This hurdle can be overcome by using, in place of styrene, *p*-*tert*-butylstyrene that allows the synthesis of polyethylene containing isolated units of the aromatic comonomer. 

The strong point of the post-metallocenes is ascribable to the wide variability of the coordinative framework, in particular the electronic feature of the atoms coordinated to the metal. So, as opposed to what observed by Scott, the OSSO complexes of titanium by Okuda et al. (Figure 1s) efficiently copolymerize these two monomers to macromolecules with long styrene homosequences [47] or even to isotactic polystyrene containing isolated ethylene units [48].

### 2.6. Other Ethylene–Styrene Polyinsertions

Now we want cite some not strictly pertinent achievements of the mechanistic studies on the catalysis of polyinsertion. First of all, it is worth mentioning the work of Dong and Chung [49] who (through the reaction of ethylene, *p*-methylstyrene, and hydrogen in presence of metallocene-based catalysts) were able to produce polyethylene chains capped by a functionalizable aromatic ring through consecutive chain transfer reaction to *p*-methylstyrene and hydrogen. The key role to achieve this result seems played by the “dormant state” of the catalytic center after styrene insertion: the metal-secondary chain is preferentially prone to the reaction with hydrogen that cleaves the bond thus reactivating the catalyst.

In a more recent paper, some of us reported how the mechanistic studies on the reactivity of different catalysts towards the title monomers suggested the idea of employing the polyinsertion to obtain relevant intermediates of the detergent industry, such as the linear alkylbenzenes. Actually, the knowledge of the regiochemistry of styrene insertion and the use of suitable feed allowed the achievement of the one-pot synthesis of linear 1-alkylbenzene from ethylene, styrene, and hydrogen [50].

## 3. Conclusions

The homogeneous polyinsertion catalysis has shown that styrene and ethylene can be enchained together to give copolymers with a wide variety of structures and, consequently, properties. From the elastomeric materials of the Dow patent (ES interpolymers), to the thermoplastic isotactic ethylene-*alt*-styrene copolymer, to the ethylene-*b*-styrene copolymer to, finally, the isotactic styrene-*b*-(ethylene-*alt*-styrene) copolymer. Behind the different ways the two monomers are distributed along the polymer chain there is the regiochemistry of insertion of the styrene that is possibly sensitive to the catalytic site geometric, electronic features, and other finer mechanistic aspects. From this point of view, the ethylene–styrene system shows how the mechanistic studies on the catalysis could disclose the way to create new polymer chain architectures through the development of the potentiality of the monomer couple.
